# Cystic Tuberculosis of the Humerus

**DOI:** 10.1590/0037-8682-0100-2022

**Published:** 2022-07-25

**Authors:** Rojbin Ceylan Tekin, Emin Özkul, Recep Tekin

**Affiliations:** 1 Mardin Training and Research Hospital, Department of Radiology, Mardin, Turkey.; 2 Dicle University, Faculty of Medicine, Department of Trauma and Orthopedic Surgery, Diyarbakir, Turkey.; 3 Dicle University, Faculty of Medicine, Department of Infectious Diseases and Clinical Microbiology, Diyarbakir, Turkey.

**FIGURE 1:**
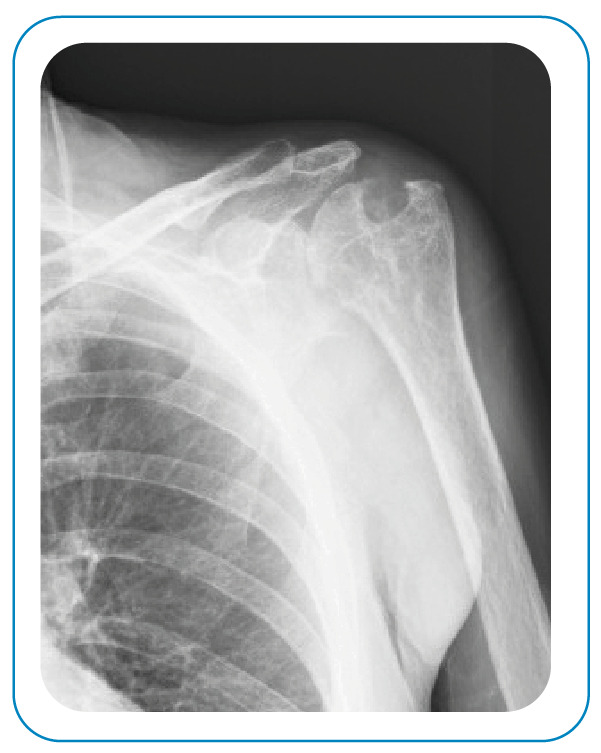
Radiological examination of the left humerus revealed osteolytic lesions.

**FIGURE 2:**
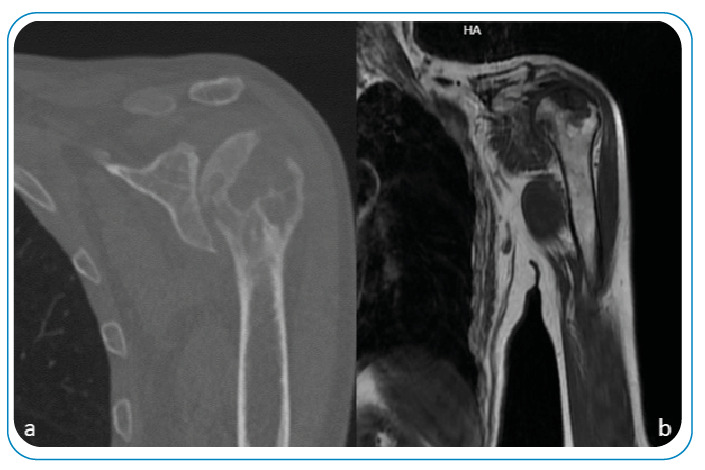
Computed tomography scan of the left shoulder showed a crescentic lucency in the humeral head **(a)** and magnetic resonance imaging of the patient’s left shoulder area revealed extensive hypointense erosions of the left humerus **(b)**.

**FIGURE 3:**
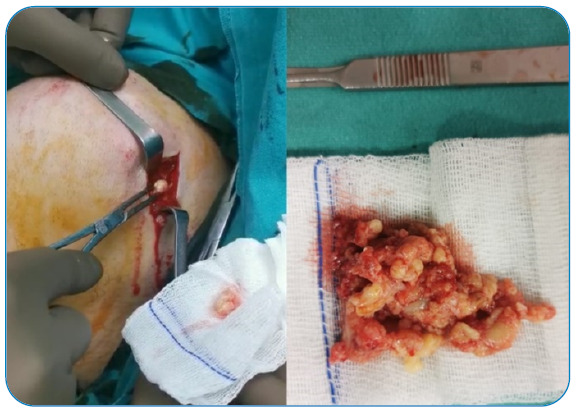
Intraoperative image showing thick whitish pus discharge from the lesion site.

A 65-year-old man presented with complaints of pain and swelling over the left scapula for 11 months. Upon examination, he had swelling on the left side in the suprascapular area, and the shoulder joint was a mildly painful movement with minimal limitation. A radiological examination of the left humerus revealed osteolytic lesions, and soft tissue showed irregular radiolucent areas in the margin (Figure 1). Magnetic resonance imaging of the patient’s left shoulder area revealed multiple T1 hypointense, T2 hyperintense lesions in the humeral head with a cortical breach, and extensive hyperintense erosions of the left humerus with soft tissues abscess (Figure 2). He underwent drainage and curettage of the swelling, caseous necrotic tissue, granulation tissue, and necrotic bone (Figure 3). Histopathology showed a chronic inflammatory process with a granulomatous reaction and caseating necrosis consistent with tuberculosis. The patient was started on four-drug anti-tuberculous chemotherapy, comprising isoniazid, rifampicin, pyrazinamide, and ethambutol. Although the primary treatment of osteoarticular tuberculosis is medical, surgery is sometimes necessary^1^ ^,^ ^2^. Tuberculosis should be considered for differential diagnosis of the adults presenting with longstanding complaints of pain and swelling in the shoulder region^3^. Unusual presentations of tuberculosis should be kept in mind to avoid delay in diagnosis and appropriate antitubercular therapy.
